# Multiple Endocrine Neoplasia with Multiple PGLs in Two Boxer Dogs: Morphological Features, Immunohistochemical Profile and SDHD Gene Mutation Screening

**DOI:** 10.3390/vetsci11110586

**Published:** 2024-11-20

**Authors:** Ecaterina Semzenisi, Roxana Popa, Corina Toma, Valentin-Adrian Bâlteanu, Iuliu Calin Scurtu, Romelia Pop, Alexandru-Flaviu Tăbăran

**Affiliations:** 1 Department of Veterinary Pathology, Faculty of Veterinary Medicine, University of Agricultural Sciences and Veterinary Medicine, 400372 Cluj-Napoca, Romania; ecaterina.semzenisi@student.usamvcluj.ro (E.S.); romelia.pop@usamvcluj.ro (R.P.);; 2 Institute of Life Sciences, University of Agricultural Sciences and Veterinary Medicine, 400372 Cluj-Napoca, Romania; lzga.usamvcl@yahoo.com; 3 Department of Internal Medicine, University of Agricultural Sciences and Veterinary Medicine, 400372 Cluj-Napoca, Romania

**Keywords:** aortic body tumor 1, carotid body tumor 2, dog 3, SDHD 4, MEN 5

## Abstract

This study aimed to describe the gross and histological features of multiple endocrine and non-endocrine neoplasia, including multiple PGLs found in two Boxer dogs. Additionally, the identified PGLs were immunohistochemically evaluated, and the subunits 2, 3, and 4 of the SDHD gene were screened for possible mutations. The tumors identified include aortic and carotid body PGLs, thyroid follicular-compact carcinoma, and subcutaneous lipomas. One case also had a Leydig cell tumor and adrenal cortex hyperplasia, while the other had H-type pancreatic carcinoma. Three out of 4 PGLs appeared benign, but one aortic body tumor showed malignant features with neoplastic emboli at its edge. Immunohistochemical analysis confirmed the neuroendocrine origin of all PGLs, with positive staining for Chromogranin A, NSE, and variable positivity for S100. No somatic mutations were found in exons 2, 3, and 4 of the SDHD gene in any of the evaluated PGLs. The absence of mutations in the evaluated SDHD gene subunits suggests the involvement of other genetic factors or pathways in the development of these tumors, warranting further investigation in this field.

## Simple Summary

Multiple Endocrine Neoplasia (MEN) is a rare genetic disorder of humans and animals, characterized by developing tumors in multiple endocrine glands. Paraganglioma (PGL) is often linked with MEN, and its occurrence is commonly associated with metabolism-related mutation genes, which currently include SDHA, SDHB, SDHC, SDHD, and SDHAF2. While sporadic cases of concurrent endocrine neoplasia have been documented in veterinary medicine, particularly in dogs, genetic investigations in this domain are lacking and still limited.

## 1. Introduction

Multiple endocrine neoplasia (MEN) refers to a group of rare inherited disorders characterized by the development of multiple endocrine and non-endocrine tumors [1,2]. In humans, several types of MEN exist, each associated with specific genetic mutations and distinct tumor types. The three main types of MEN are MEN1, MEN2A, and MEN2B, although additional subtypes have also been proposed. The classification criteria for multiple endocrine neoplasia (MEN) syndromes are based on distinct patterns of tumors. MEN1 involves tumors in the parathyroid glands, endocrine pancreas, and anterior pituitary. MEN2A is characterized by medullary thyroid carcinoma, pheochromocytoma, and parathyroid hyperplasia, while MEN2B includes more aggressive medullary thyroid carcinoma, pheochromocytoma, and neuromas but lacks primary hyperparathyroidism [3,4,5,6,7].

MEN-like syndromes have been described in veterinary medicine as well, though they are relatively rare compared to their occurrence in humans and are poorly documented in terms of both oncological presentation and genetic causes [8,9,10,11,12,13,14]. In dogs, the most commonly observed endocrine tumors are adrenal, thyroid, and pancreatic tumors [15]. Cats can also develop endocrine tumors, primarily thyroid tumors (such as thyroid adenomas or carcinomas) and, less frequently, pancreatic tumors [16]. In cattle and horses, the most common endocrine tumor is pituitary adenoma, though thyroid and adrenal neoplasms have also been reported sporadically. In small ruminants, endocrine tumors are rarely reported [17,18,19,20]. Among animals, MEN-like syndromes have been most frequently documented in dogs.

From a genetic perspective, the development of paragangliomas and pheochromocytomas can be categorized into two primary clusters. The first cluster is characterized by a pseudohypoxic signature involving genes related to the tricarboxylic acid (TCA) cycle, such as SDH (succinate dehydrogenase), VHL (von Hippel-Lindau), and FH (fumarate hydratase). The second cluster is defined by a kinase signature, including genes like RET (rearranged during transfection), NF1 (neurofibromin 1), and TMEM127 (a gene that encodes a membrane protein, mutations that are associated with pheochromocytomas. It functions as a tumor suppressor), and MAX (that gene encodes a transcription factor involved in cell proliferation and apoptosis). In humans, tumors in this kinase-driven cluster often include pheochromocytomas and paragangliomas associated with multiple endocrine neoplasia type 2 (MEN2) and neurofibromatosis type 1 (NF1) [6,7,21].

Paraganglioma-pheochromocytoma syndrome (PGL-PCC) is a distinct genetic disorder with a broad spectrum of hereditary predispositions and should be differentiated from MEN2, a separate genetic disorder caused by mutations in the RET proto-oncogene. MEN2 is typically associated with tumors in the thyroid gland (often medullary thyroid carcinoma), parathyroid glands (primary hyperparathyroidism), and adrenal glands (pheochromocytomas). In contrast, PGL-PCC is not linked to RET mutations but rather to mutations in SDH subunit genes [6,22,23].

In veterinary medicine, there remains a significant gap in comprehensive genetic databases that could provide detailed insights into tumorigenesis, similar to those available in human medicine. Recently, a mutation in the pseudohypoxic cluster related to SDH was identified in dogs, suggesting an association with MEN-like syndromes [24,25].

The succinate dehydrogenase complex subunit D (SDHD) gene is located on chromosome 11q23 and encodes a protein that is a key component of mitochondrial complex II, which functions within the electron transport chain. Mutations in the SDHD gene disrupt this protein’s function, leading to dysregulation of cellular metabolism and enhanced cellular proliferation [26,27].

In the context of paraganglioma-pheochromocytoma syndrome (PGL-PCC), paragangliomas (PGLs) are neuroendocrine tumors arising from paraganglia, which consist of clusters of neuroendocrine cells. PGLs develop outside the adrenal glands, originating from either the sympathetic or parasympathetic paraganglia [28]. These neuroendocrine cells, also known as chromaffin or chemoreceptor cells, are sensitive to changes in blood carbon dioxide, oxygen levels, and pH, playing critical roles in regulating respiration and circulation. PGLs can occur in various regions of the body, including the head, neck, thorax, abdomen, and pelvis [29,30,31].

In brachycephalic dogs, PGLs of the carotid and aortic bodies are more frequently observed, likely due to a chronic hypoxic state [25,30,32]. Despite this observation, few genetic studies have been conducted on endocrine tumors in dogs. A somatic missense mutation in exon 4 (c.365A>G) of the SDHD gene, causing a p.Lys122Arg substitution in the protein, was identified in canine PGLs [24,25] screening exons 2, 3, and 4 of the SDHD gene in canine paragangliomas (PGL) and pheochromocytomas (PC) is crucial due to potential role in tumorigenesis. Both Holt and Korpershoek’s studies emphasize that mutations in these exons, particularly in exon 4, are often associated with loss of heterozygosity (LOH) in tumor samples. This observation aligns with the two-hit hypothesis, suggesting that SDHD may act as a tumor suppressor gene, where both alleles must be inactivated for tumor formation. Exon 4 specifically was identified as a mutation hotspot, with a missense mutation (c.365A>G, p.Lys122Arg) that was also accompanied by LOH, indicating a significant role in the disruption of the gene’s tumor-suppressing function. Additionally, exons 2 and 3, though less frequently implicated, showed mutations that could potentially influence RNA splicing and stability, further supporting the relevance of these regions in tumor development. This study aims to (1) describe the gross and histological features of multiple endocrine and non-endocrine neoplasms identified in two Boxer dogs; (2) analyze the immunohistochemical profile of the identified PGLs; and (3) sequence exons 2, 3, and 4 of the SDHD gene to assess for possible mutations linked to PGL development.

## 2. Materials and Methods

### 2.1. Sample Collection and Histological Processing

A 10-year-old male (Case 1) and a 12-year-old female (Case 2) Boxer were submitted for postmortem examination at the Department of Veterinary Pathology, Faculty of Veterinary Medicine, Cluj-Napoca. Both dogs had a history suggestive of congestive heart failure, and a heart base neoplasm was suspected in each based on ultrasound findings. No information is available regarding the pedigree of the two Boxer dogs, including whether they were siblings or closely related through a shared bloodline. 

Samples were collected in duplicate from normal tissues (adipose tissue and muscle) and tumor tissues (from the carotid artery and aorta). For genetic analysis, samples were transferred into sterile Eppendorf tubes and stored at −20 °C for subsequent DNA extraction to analyze SDHD gene polymorphisms. Additional tumor tissues were placed in 50 mL tubes for histopathological and immunohistochemical preparation.

All collected samples were fixed in 10% formalin for at least 48 h for histological analysis, followed by routine paraffin embedding. After fixation, the samples were dehydrated through a series of ethanol solutions (80%, 95%, and 100%) to remove water. The tissues were cleared using xylene, which made them transparent and prepared them for embedding in paraffin. The paraffin blocks were sectioned into 2 µm sections and either stained with hematoxylin and eosin (H&E) or stored for later immunohistochemical analysis. The slides were evaluated under an Olympus BX-41 light microscope, and photomicrographs were captured using an Olympus SP 350 digital camera linked to Stream Basic imaging software (Olympus Corporation, Tokyo, Japan).

### 2.2. Immunohistochemistry

For immunohistochemical assessment, Chromogranin A, Neuron Specific Enolase (NSE), and S100 protein antibodies were selected (Table 1). The tissue processing for Immunohistochemistry (IHC) begins with sectioning the tissue and placing it on specially treated slides to improve tissue adhesion. The slides are then heated at 60 °C to enhance the attachment of tissue sections. All specimens underwent automated processing utilizing the Leica Bondmax TM Immunohistochemistry system (Leica Biosystems Melbourne, Bond Max model, M2 12,154 series). Positive reactions were identified by the brown staining of the cytoplasm for Chromogranin A (Cr-A) and NSE, as well as both cytoplasm and nuclei for S100. Positive controls were included for each antibody. Negative controls were obtained by substituting the primary antibody with serum.

### 2.3. Genetic Analysis of SDHD Gene Polymorphism in Healthy and Neoplastic Tissue

Genomic DNA was purified from 10 mg from each fresh-frozen sample collected from neoplastic (carotid and aortic tumors) and healthy tissues (muscle and adipose tissue) of two Boxer dogs, using the Isolate II Genomic DNA kit (Meridian Bioscience, London, UK), according to manufacturer instructions. The quantification of DNA was assessed using a NanoDrop ND-100. The samples were diluted to a final concentration of 50 ng/µL.

For PCR amplification, three primer sets earlier described by Holt et al., 2014 were used to amplify the entire sequences and part of the flanking regions of the exons 2, 3, and 4 of the SDHD gene [24], as shown in Table 2. 

The PCR amplification of three exons from the SDHD gene from tumor and normal tissues was performed using the 2X MyTaq™ Mix (Meridian Bioscience, London, UK). Each reaction was made up of 25 μL final volume, using 12.5 μL 2X MyTaq, diluted with 9.5 µL of ultrapure water, 1 μL (10 pmol) of each primer (depending on the amplified region), and 1 μL (50 ng) of genomic DNA. PCR amplification was carried out in a CFX96 thermocycler (Bio-Rad, Hercules, CA, USA). The PCR was carried out using the following cycling profile: 94 °C for 5 min, one cycle and at 94 °C for 1 min, 60 °C for 1 min, and 72 °C for 1 min for 35 cycles. To avoid obtaining unspecific amplicons, we modified the original annealing temperature to amplify exons 2 and 3 described by Holt et al., 2014 [24] from 54 to 60 °C. A volume of 6 μL from each PCR reaction was examined for amplification specificity on a 2% agarose gel stained with 1X SybrSafe (Invitrogen, Waltham, MA, USA) electrophoresed in 1X TBE buffer.

The PCR amplicons were sequenced with the BigDye™ Terminator v3.1 Cycle Sequencing Kit with the same PCR primers. The sequencing products were purified and, after formamide denaturation, were analyzed on SeqStudio equipment (Thermo Fisher Scientific, Waltham, MA, USA).

The sequencing chromatograms obtained from healthy and neoplastic tissues were comparatively analyzed using the Chromas (Source: http://www.technelysium.com.au/wp/chromas/) accessed on 15 November 2023.

## 3. Results

### 3.1. Gross Examination

**Figure 1:**
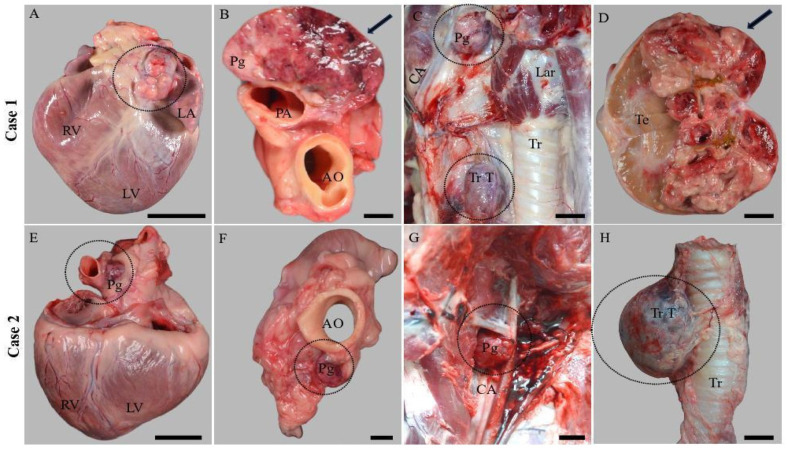
Gross features of the multiple endocrine neoplasias identified in the two dogs. (**A**) Aortic body tumor (dotted circle) exhibiting an infiltrative growth pattern; scale bar: 5 cm. (**B**) Aortic body tumor (arrow); on cross-section, the neoplastic mass was multilobular, grayish-yellow color, and admixed with areas of hemorrhages; scale bar: 1 cm. (**C**) Thyroid neoplasia and carotid body tumor (dotted circles); scale bar: 1 cm. (**D**) Right testicle partially replaced by a multilobular friable neoplastic mass (arrow) with gross features of Leydig cell tumor; scale bar: 2 cm. (**E**) Aortic body tumor (dotted circle), a 1–2 cm well-delimited neoplastic mass identified between the aorta and pulmonary trunk (dotted circle); scale bar: 4 cm. (**F**) Apical view of the aortic body tumor (dotted circle); scale bar: 1 cm. (**G**) Carotid body tumor (dotted circle) located at the bifurcation of the common carotid artery; scale bar: 1 cm. (**H**) The right thyroid gland was expanded and replaced by a well-delimited, round-to-oval encapsulated neoplastic mass; scale bar: 1 cm. RV: right ventricle; LA: left auricle; LV: left ventricle; PA: pulmonary artery; AO: aorta; Tr T: thyroid tumor; Lar: larynx, CCA: common carotid artery; Te: testicle; CA: carotid artery; and Pg: PGL.

Case 1: A 10-year-old male Boxer was presented for necropsy. Gross examination revealed a multilobular, encapsulated, and dense neoplastic mass measuring 5–6 cm in diameter, located at the base of the heart between the pulmonary trunk and aorta. The mass exhibited an infiltrative growth pattern, presenting a mosaic appearance on cross-section, with grayish-yellow regions interspersed with reddish hemorrhagic areas. Additionally, a similar neoplastic mass, approximately 2 cm in diameter, was identified at the bifurcation of the left common carotid artery, suggestive of a paraganglioma/chemodectoma (Figure 1). The right thyroid gland was enlarged, with a well-demarcated neoplastic mass measuring 4 cm in diameter, partially replacing the gland. Examination of the reproductive organs revealed a friable, yellow-reddish neoplastic mass, measuring 5–6 cm in diameter, in the right testicle, consistent with a Leydig cell tumor. The cortex of the right adrenal gland was partially replaced by a well-defined yellowish nodule 0.5 cm in diameter. Skin and subcutis examination identified multiple well-demarcated subcutaneous neoplastic masses, consistent with lipomas based on gross morphology. Additional findings included signs of chronic liver congestion, pleural effusions, and ascites.

Case 2: A 12-Year-Old Female Boxer was referred for necropsy. A multilobular, encapsulated, dense neoplastic mass measuring 1–2 cm in diameter was observed at the base of the heart, between the aorta and the pulmonary trunk. The mass was well delimited with a heterogenous pattern on cross-section, with grayish-yellow areas and hemorrhagic areas. An approximately similar neoplastic mass of 2 cm in diameter was found at the bifurcation of the left common carotid artery, and it was suspected to be a paraganglioma/chemodectoma. The right thyroid gland was enlarged and partially replaced by a well-delimited neoplastic mass measuring 5 cm in diameter. An infiltrative pancreatic neoplasm was identified, and it was associated with peritoneal carcinomatosis. Multiple well-delimited subcutaneous neoplastic masses with gross features of lipomas were also present. Other findings included signs of chronic liver congestion and ascites.

### 3.2. Histological Examination

**Figure 2:**
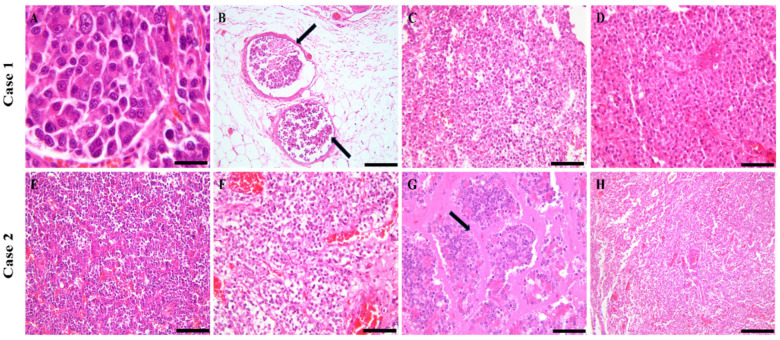
Histological features of the endocrine and non-endocrine neoplastic masses were identified in the two cases. (**A**) Aortic body PGL; the neoplastic cells were round to polygonal in shape, exhibiting mild anisokaryosis and anisocytosis; 40×, scale bar: 200 µm. (**B**) Multiple neoplastic emboli (arrows) were identified within the lymphatic vessels of the epicardial adipose tissue; 20×, scale bar: 200 µm. (**C**) Carotid body PGL; the neoplastic cells were arranged in nests, separated by a fine fibrovascular stroma; 20×, scale bar: 200 µm. (**D**) Leydig cell tumor; 20×, scale bar: 200 µm. (**E**) Aortic body PGL. (**F**) Carotid body PGL; 20×, scale bar: 200 µm. (**G**) Pancreatic adenocarcinoma composed of neoplastic cells predominantly organized as acini and delimited by abundant acidophilic, homogenous, hyaline material (arrow); 20×, scale bar: 200 µm. (**H**) Thyroid carcinoma; 40×, scale bar: 200 µm.

Case 1: The histopathological examination of both aortic and carotid body revealed well-cellularized neoplastic masses composed of round to polygonal neoplastic cells arranged in nests and packets, separated by a delicate fibrovascular stroma, characteristic of a “Zellballen” pattern. The neoplastic cells displayed distinct cell membranes, moderate amounts of finely granular, lightly acidophilic cytoplasm, and round to oval nuclei with finely stippled chromatin and 1–3 prominent nucleoli. Multiple neoplastic emboli were identified within lymphatic vessels in adjacent adipose tissue. Mitoses were rare, observed at a rate of 0–1 per 2.37 mm^2^. In the thyroid gland, an infiltrative neoplastic mass was noted, composed of cuboidal to polygonal cells arranged into follicles and papillary structures, also separated by a fine fibrovascular stroma. These cells contained moderate to abundant, deeply acidophilic cytoplasm and vesicular nuclei with prominent nucleoli. Occasional cellular pleomorphism and mitotic figures were observed. These features are consistent with follicular-compact (or mixed) thyroid carcinoma. In the testicular mass, the normal parenchyma was replaced by neoplastic cells organized in nests and packets, delimited by a delicate fibrovascular stroma. The neoplastic cells exhibited abundant, acidophilic, finely granular cytoplasm with occasional lipochrome pigment. The nuclei were round, hyperchromatic, and parabasal, with 1–2 distinct nucleoli. Minimal anisocytosis and anisokaryosis were present, with rare mitotic figures (Leydig cell tumor). Histological examination of the adrenal gland mass revealed a nodular pattern of cortical cells within the medulla, indicative of cortical-medullary hyperplasia Figure 2.

Case 2: The histological features of the aortic and carotid body masses were similar to those observed in Case 1, consisting of densely cellular neoplastic masses with round to polygonal cells arranged in nests and packets, exhibiting the characteristic “Zellballen” pattern. Anisocytosis and anisokaryosis were less pronounced compared to Case 1. Within the thyroid gland, an infiltrative neoplastic mass was identified, composed of cuboidal to polygonal cells organized into follicles and papillary structures, separated by a delicate fibrovascular stroma. The cells exhibited moderate to abundant deep acidophilic cytoplasm and vesicular nuclei with prominent nucleoli. Occasional cellular pleomorphism and mitotic figures were observed. These features are consistent with follicular-compact thyroid carcinoma. The pancreatic mass was composed of acinar and tubular structures, surrounded by lakes and aggregates of acidophilic hyaline material. Neoplastic cells were cuboidal to polygonal, with abundant eosinophilic, granular cytoplasm localized at the apical ends. The nuclei were basally positioned, round to oval, hyperchromatic, with prominent nucleoli and rare mitotic figures. Mild anisocytosis and nuclear pleomorphism were present. Occasionally, the hyaline material embeds nests or cords of shrunken, atrophic neoplastic cells, which is consistent with hyalinizing pancreatic carcinoma Figure 2. 

### 3.3. Immunohistochemical Examination

In the first case, the neoplastic cells in both the aortic and carotid body masses exhibited mild to moderate cytoplasmic immunolabeling for Chromogranin-A (Cr-A), indicated by granular brown staining. A diffuse and intense cytoplasmic immunoreactivity was also observed for Neuron-Specific Enolase (NSE) in both masses. For S100, the aortic body paraganglioma (PGL) showed intense immunolabeling, whereas the carotid body PGL displayed only weakly positive immunostaining (Table 3).

**Figure 3:**
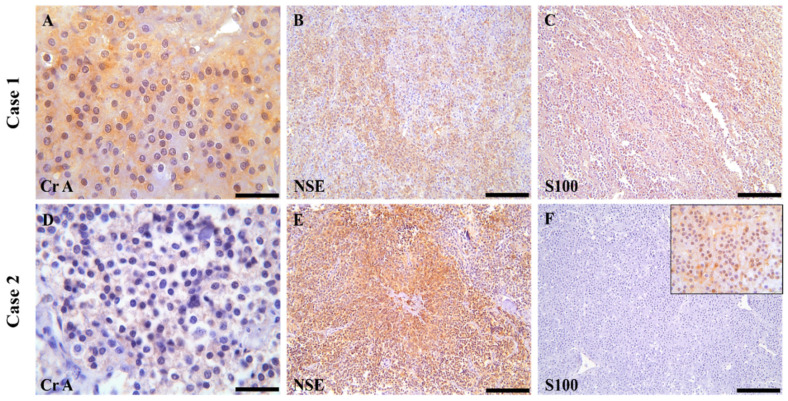
Immunohistochemical features of the aortic and carotid body tumors identified in two dogs. (**A**) Aortic body tumor; 40×, scale bar: 200 µm and (**D**) Carotid body tumor; 20×, scale bar: 200 µm were both multifocally and variably positive for Cr-A. (**B**) Carotid body tumor and (**E**) Aortic body tumor; both were 20×, scale bar: 200 µm. The neoplastic cells were multifocally immunopositive for NSE. (**C**,**F**) Images of S100 immunoexpression: (**C**) Case 1; the S100 protein immunoexpression was diffusely present for the carotid body tumor; 20×, scale bar: 200 µm; and (**F**) Case 2; the aortic body tumor was immunonegative for S100 and weakly positive for the carotid body tumor (inset); 20×, scale bar: 200 µm.

In the second case, the immunohistochemical analysis of the aortic and carotid body paragangliomas revealed multifocal mild to moderate positivity for Chromogranin-A (Cr-A), with neoplastic cells exhibiting granular brown cytoplasmic staining. In both anatomical locations, there was a multifocal to diffuse and intense cytoplasmic immunoreaction for Neuron-Specific Enolase (NSE), highlighting the presence of neuroendocrine differentiation. However, the immunostaining results for S100 differed between the two sites; the aortic body PGL showed negative immunostaining, while the carotid body tumor exhibited diffuse but weak positivity Figure 3. In assessing thyroid follicular carcinomas, Chromogranin-A (Cr-A) revealed multifocal and variable positivity in both cases, indicating a neuroendocrine component. The neoplastic cells were also multifocally intensely positive for Neuron-Specific Enolase (NSE), further supporting the neuroendocrine characteristics of the tumors. In contrast, S100 showed negative results across the examined specimens Additional data can be found in the Supplementary Materials Figure S1.

### 3.4. Amplification and Sequencing of Exons 2, 3, and 4 of the SDHD Gene from Healthy and Tumoral Tissues

The electrophoretic analysis of the PCR products obtained from exons 2, 3, and 4 of the SDHD gene evidenced the presence of specific amplicons of 227, 263, and 381 bp, respectively.

The comparative analysis of the chromatograms containing the full sequence of the SDHD gene exon 2, exon 3, and exon 4 obtained aortic and carotid bodies tumors versus healthy tissue, i.e., muscle, did not reveal any somatic mutations in either of the two Boxer dogs, with a nucleotide similarity of 100% throughout the entire encoding sequences.

**Figure 4:**
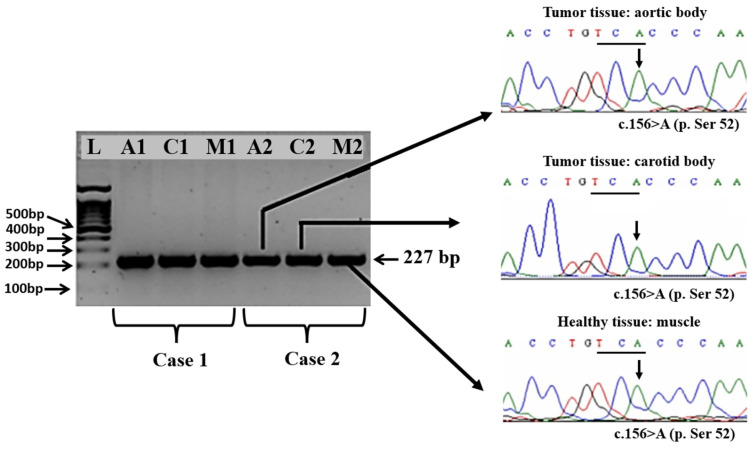
Specific amplicons obtained by amplification of the exon 2 of the *SDHD* gene from tumor tissues, i.e., aortic body (A1 and A2), carotid body (C1 and C2), and healthy tissue, i.e., muscle (M1 and M2) collected from the two Boxer dogs and part of the sequencing chromatograms evidencing the presence of wild type sequence, in particular at the position c.156 A.

In particular, we found that the synonymous wildtype mutation from exon 2 at position c.156A from the codon 52-TCA encoding for p. 52Ser was present in both healthy tissues and tumoral tissues (Figure 4).

**Figure 5:**
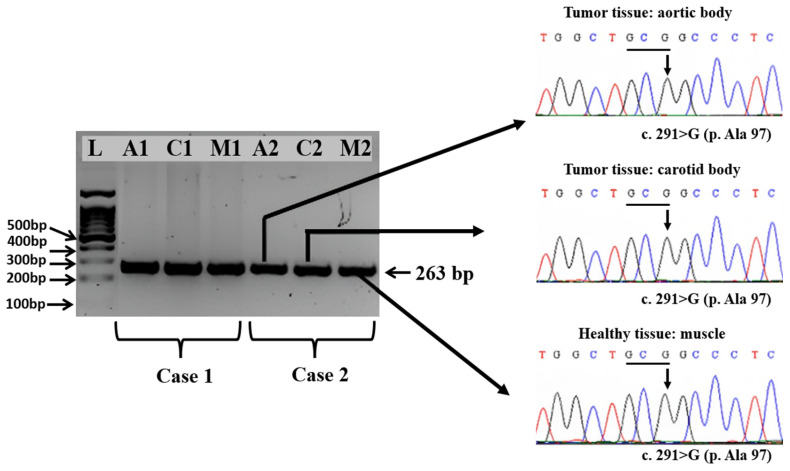
Specific amplicons obtained by amplification of the exon 3 of the *SDHD* gene from tumor tissues, i.e., aortic body (A1 and A2), carotid body (C1 and C2), and healthy tissue, i.e., muscle (M1 and M2) collected from the two Boxer dogs and part of the sequencing chromatograms evidencing the presence of wild type sequence, in particular at the position c.291 G. Amplicons specific to exon 4 of the SDHD gene were generated from tumor tissues, including the aortic body (A1 and A2) and carotid body (C1 and C2), as well as from healthy muscle tissue (M1 and M2) sampled from the two Boxer dogs. Sequencing chromatograms confirmed the presence of the wild-type sequence, notably at position c.365 A (Figure 6).

At the same time, in exon 3, we identified in both types of tissues the presence of wild-type synonymous mutation at position c.291G from the codon 97-GCG encoding for p. 97Ala (Figure 5).

The summary of the obtained results is presented in Table 3.

## 4. Discussion

To date, there are no documented or confirmed screenings for hereditary syndrome genes in veterinary medicine that resemble multiple endocrine neoplasia (MEN) as observed in humans. In human patients with paragangliomas, those carrying SDHD germline mutations face a significantly higher risk of developing distant metastases, making this mutation a critical indicator of potential malignant progression. Animal models are vital in advancing diagnostic and therapeutic strategies for human paragangliomas.

Two previous studies [24,25] investigating the role of the SDHD gene in canine endocrine neoplasias demonstrated that a synonymous mutation (c.365A>G, p.Lys122Arg) in dogs is associated with the presence of paragangliomas (PGL) and pheochromocytomas (PCs). In the absence of rodent models of paraganglioma with SDHD mutations, the presence of this mutation in dogs, which mirrors the human condition, is particularly significant from a comparative pathology perspective. In these studies, Holt et al. (2014) found that 2 out of 6 PCCs and 1 out of 2 PGLs had SDHD mutations [24]. Similarly, in the study by Korpershoek et al. (2019) [25], 3 out of 32 PCCs and 1 out of 18 PGLs were found to carry SDHD mutations. Our study’s data and results indicate that not all cases of paragangliomas associated with other neoplasms will exhibit mutations in the SDHD gene. Alternative genetic drivers or mutations in other genes may contribute to developing paragangliomas and pheochromocytomas (PPGLs). The absence of SDHD mutations in some canine PPGLs could be attributed to the presence of these alternative genetic factors. Additionally, technical limitations and tumor heterogeneity may impact mutation detection, potentially resulting in the omission of relevant variants in certain tumor areas or samples. Consequently, the genetic background of paragangliomas in dogs appears to be much more complex [21].

While instances of multiple neoplastic and/or hyperplastic lesions within the endocrine organs have been documented in dogs, such associations remain rare [32]. Endocrine tumors are infrequent in this species, accounting for approximately 1–2% of all diagnosed tumors [33]. Among this spectrum, paragangliomas represent only 0.2% of all canine tumors [34]. Brachycephalic breeds, such as Boxers, are considered to have an elevated risk of developing paragangliomas, with chronic hypoxia suggested as a potential contributing factor [35]. The primary sites where paragangliomas typically develop include the aortic body, located between the aorta and the pulmonary trunk, and the carotid body, found at the bifurcation of the common carotid artery [36,37].

In dogs, aortic body paragangliomas (PGLs) are typically benign, unlike carotid body PGLs. These tumors exhibit slow growth through expansion, leading to increased pressure in the vena cava and atria. Rarely, aortic body PGLs may invade the pericardium and adjacent blood vessels [38]. Malignant neoplasms of the aortic body are less common in dogs than benign forms. They can infiltrate the pulmonary artery wall through papillary projections into the blood vessel lumen or invade the atrial lumen. Although these tumors frequently invade blood vessels, pulmonary or hepatic metastases occur inconsistently [38,39,40].

In both cases described here, multiple tumors affecting both endocrine and non-endocrine organs were identified. In Case 1, alongside the PGLs, additional findings included endocrine tumors of the testicle and thyroid gland and a nodular area of hyperplasia within the cortex of one adrenal gland. In Case 2, in addition to the PGLs, tumors of the thyroid gland, pancreas, and multiple lipomas were identified. Three of the four identified PGLs displayed gross and histological features characteristic of benign tumors. No metastases were detected in any of the examined tumors. However, in Case 1, the aortic body PGL exhibited an infiltrative growth pattern, and multiple lymphatic neoplastic emboli were found in the surrounding tissues, indicating characteristics consistent with a malignant PGL.

Distinguishing between benign and malignant paragangliomas is challenging, and there is currently no established consensus on how to make this distinction, particularly from a clinical perspective. Pathologically, malignant PGLs have been shown to present a different antigenic profile than benign ones, which may be attributed to alterations in the mechanisms involved in the biosynthesis of secretory granules [41,42]. In their study, the tumor grade was inversely proportional to the immunohistochemical expression for Chromogranin A and S-100. According to Aresu [1], antibodies targeting Chromogranin A and S-100 are useful markers for this differentiation, as well as for assessing tumor grade and prognosis concerning histological criteria. The results for Chromogranin A and S-100 were negative in poorly differentiated tumors and metastases. Specifically, S-100, which labels supporting cells, showed that in malignant tumors, results correlated with a loss of solid structure and a reduced density of these cells. However, in the second case, the aortic body PGL tested negative for S-100, suggesting a potentially malignant nature, although there were no gross histological features indicative of malignancy. One plausible explanation for this discrepancy in the second case could be the presence of metastases that were not visibly apparent during macroscopic examination [9,42].

None of the mutations reported in Holt et al.’s (2014) [24] study was identified in the PGLs of the two Boxer dogs studied. In exon 4, both healthy and neoplastic tissues exhibited the wild-type nucleotide c.365A from the AAA codon (p.Lys122), which contrasts with the findings reported in the literature [24,25]. These results highlight the complexity of the genetic basis of PGL development in dogs, indicating that the causative mutations may reside in other gene clusters. To fully understand the pathogenesis of paragangliomas, future research should focus on screening both clusters of gene expression: the hypoxic/angiogenic cluster and the kinase-signaling cluster. 

While the study provides valuable initial insights into the presence of multiple endocrine tumors in Boxer dogs, the small sample size (limited to two cases) presents a limitation in terms of generalizability. As these cases may not fully represent the prevalence and genetic underpinnings of multiple endocrine tumors in the broader Boxer population or other canine breeds, future research with larger, more diverse sample sizes is necessary to confirm these findings and explore their applicability to the wider canine population. Expanding the sample size in subsequent studies could also help uncover potential breed-specific genetic predispositions and variations in tumor presentation, offering a more comprehensive understanding of endocrine tumor prevalence and related genetic factors in dogs.

## 5. Conclusions

A comprehensive investigation into multiple endocrine neoplasia, including aortic body and carotid body paragangliomas (PGLs), was conducted in two Boxer dogs. The associated endocrine tumors identified included thyroid mixed carcinoma, Leydig cell tumors, pancreatic carcinomas, and multiple subcutaneous lipomas. While three out of four PGLs exhibited characteristics typical of benign tumors, one aortic body tumor displayed malignant features. Analysis of the nucleotide sequences of exons 2, 3, and 4 of the SDHD gene from both neoplastic and healthy tissues of the same dog revealed no alterations. These findings highlight the complexity of the genetic factors underlying PGL occurrence in dogs, suggesting that its development may also be influenced by mutations in other genes.

## Figures and Tables

**Figure 6:**
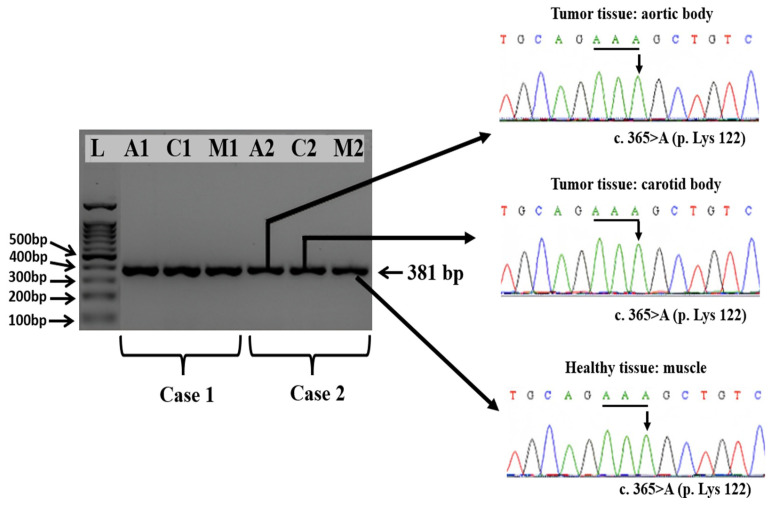
Specific amplicons obtained by amplification of the exon 4 of the *SDHD* gene from tumor tissues, i.e., aortic body (A1 and A2), carotid body (C1 and C2), and healthy tissue, i.e., muscle (M1 and M2) collected from the two Boxer dogs and part of the sequencing chromatograms evidencing the presence of wild type sequence, in particular at the position c.365 A.

**Table 1:** List of antibodies used for immunohistochemical evaluation.

Antibody	Clone	Manufacturer	Dilution	Positive Control
**Chromogranin A**	5H7	Leica Biosystems Newcastle Ltd. Newcastle upon Tyne, UK	Ready to use	Endocrine pancreas
**NSE**	22C9	Leica Biosystems Newcastle Ltd. Newcastle upon Tyne, UK	Ready to use	Brain
**S100**	Polyclonal	Leica Biosystems Newcastle Ltd. Newcastle upon Tyne, UK	Ready to use	Adipose tissue

**Table 2:** Primers used for PCR amplification of exons 2, 3, and 4 of the SDHD gene and the corresponding annealing temperatures (F: forward; R: reverse).

Exon	Primers	Amplicon Size	Annealing Temperature
2	2F: 5′-tgtcaggcctgttaaaagagaa-3′ 2R: 5′-cagatgtagagggccagagc-3’	227 bp	60 °C
3	3F: 5′-atgtgtgtttccccctttca-3′ 3R: 5′-atgagacaggctcacagcaa-3′	263 bp	60 °C
4	4F: 5′-tattactaggaaactcatagcccct-3′ 4R: 5′-gctgaagggcatgatagagc-3′	381 bp	60 °C

**Table 3:** Histological features, immunohistochemical profile, and genetic analyses of the SDHD gene in the studied PGLs.

Case	Histological Features of PGLs	Immunohistochemical Profile	SDHD Gene
Case 1	Round to polygonal neoplastic endocrine cells arranged in the characteristic “Zellballen” pattern and separated by a fine fibrovascular stroma	PgA-Cr A(+); S 100(+), NSE (+)	
		PgC-Cr A(+); S 100(+/−), NSE (+)	Exon 2: WT
Case 2		PgA-Cr A(+); S 100(−), NSE (+)	Exon 3: WT
		PgA-Cr A(+); S 100(+), NSE (+)	Exon 4: WT

## Data Availability

The data supporting this study are available from the corresponding author upon reasonable request.

## Supplementary Materials

The following are available online at https://www.mdpi.com/article/10.3390/vetsci11110586/s1, Figure S1: Immunohistochemical features of the thyroid follicular carcinomas. (A,D) Cr A in both cases were multifocally positive. (B,E) The neoplastic cells were multi-focally to diffuse intensely positive for NSE. (C,F) S100–Negative.

## List of Abbreviations

TCA, Tricarboxylic Acid; SDH, Succinate Dehydrogenase; SDHD, Succinate Dehydrogenase Complex Subunit D; VHL, von Hippel-Lindau; FH, Fumarate Hydratase; RET, Rearranged During Transfection; NF1, Neurofibromin 1; TMEM127, Transmembrane Protein 127; MAX, MYC Associated Factor X; MEN1, Multiple Endocrine Neoplasia Type 1; MEN2A, Multiple Endocrine Neoplasia Type 2A; MEN2B, Multiple Endocrine Neoplasia Type 2B; PGL, Paraganglioma; PCC, Pheochromocytomas; PGL-PCC, Paraganglioma-Pheochromocytoma Syndrome; H-type, Hyaline; NSE, Neuro Specific Enolase; S-100, Immunohistochemistry Staining; Cr-A, Chromogranin A; H&E, Hematoxylin and Eosin; PCR, Polymerase Chain Reaction; RV, Right Ventricle; LA, Left Auricle; LV, Left Ventricle; PA, Pulmonary Artery; AO, Aorta; Tr T, Thyroid Tumor; Lar, Larynx; CCA, Common Carotid Artery; Te, Testicle; CA, Carotid Artery; DNA, Deoxyribonucleic Acid; IHC, Immunohistochemistry.
