# Supplementary material for: Multiple Endocrine Neoplasia with Multiple PGLs in Two Boxer Dogs: Morphological Features, Immunohistochemical Profile and SDHD Gene Mutation Screening
Source: Vet Sci. 2024 Nov 20;11(11):586. doi: 10.3390/vetsci11110586 (PMC11598997; doi:10.3390/vetsci11110586)
Supplement: Supplementary file 1 [file vetsci-11-00586-s001.zip › vetsci-3234561-supplementary.pdf]

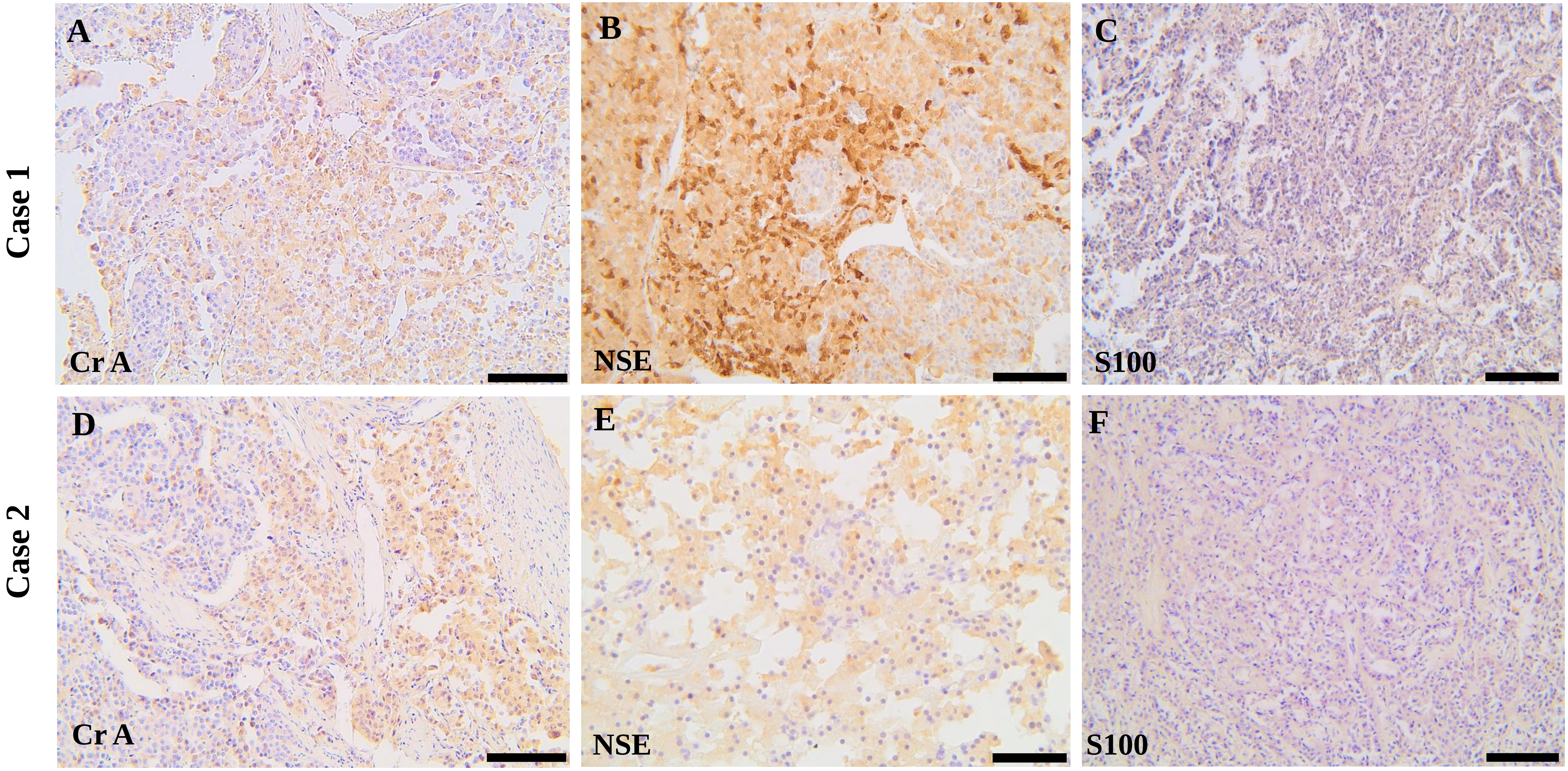

Immunohistochemical features of the thyroid follicular carcinomas. (A, D) Cr A in both cases were multifocally positive**. (B, E) T**he neoplastic cells were multifocally to diffuse intensely positive for NSE**.** **(C, F)** S100 - Negative**.**
